# Glycemic Control during Coronary Artery Bypass Graft Surgery

**DOI:** 10.5402/2012/292490

**Published:** 2012-11-14

**Authors:** Harold L. Lazar

**Affiliations:** Department of Cardiothoracic Surgery, The Boston Medical Center and The Boston University School of Medicine, 88 East Newton Street, Boston, MA 02118, USA

## Abstract

Hyperglycemia, which occurs in the perioperative period during cardiac surgery, has been shown to be associated with increased morbidity and mortality. The management of perioperative hyperglycemia during coronary artery bypass graft surgery and all cardiac surgical procedures has been the focus of intensive study in recent years. This report will paper the pathophysiology responsible for the detrimental effects of perioperative hyperglycemia during cardiac surgery, show how continuous insulin infusions in the perioperative period have improved outcomes, and discuss the results of trials designed to determine what level of a glycemic control is necessary to achieve optimal clinical outcomes.

## 1. Introduction

The incidence of diabetes mellitus in patients undergoing coronary artery bypass graft (CABG) surgery continues to increase and it is now estimated that nearly 30–40% of CABG patients will have diabetes mellitus or the metabolic syndrome [1]. Patients with diabetes mellitus have had worse outcomes following CABG [2–4]. They have higher mortality and a higher incidence of renal failure, stroke, sternal wound infections, and increased need for inotropic support [5–8]. Their length of stay is prolonged and hospital costs are increased [9]. Furthermore, diabetic CABG patients are more likely to require a repeat revascularization procedure, have a 24% higher risk of readmission for cardiac-related issues, and a 44% higher risk for rehospitalization for any cause [10, 11]. These outcomes were thought to be irreversible since diabetic patients have more diffuse coronary disease, abnormal fibrinolytic and platelet function and impaired endothelial function which leads to lower graft patency and ultimately increased perioperative mortality, reduced long-term survival, and less freedom from recurrent ischemic events [12–15]. In this paper, we will show that by achieving glycemic control in patients with diabetes mellitus undergoing CABG surgery, perioperative morbidity and mortality can be reduced, long-term survival improved, and the incidence of recurrent ischemic events decreased. 

## 2. Detrimental Effects of Hyperglycemia on the Cardiovascular System

In order to understand the beneficial effects of glycemic control during CABG surgery in patients with hyperglycemia, it is important to understand the detrimental effects of hyperglycemia on the cardiovascular system.

In the nonischemic myocardium, the primary energy substrate is free fatty acids [16]. However, during ischemia when free fatty acids cannot be metabolized, increased levels of free fatty acids can be detrimental to the myocardium because they increase oxygen consumption, depress contractility, and increase arrhythmias and oxygen free radicals, which ultimately impair endothelial function [17]. Increased levels of free fatty acids also impair glucose metabolism which is the preferred myocardial substrate during periods of ischemia [16]. In the nondiabetic ischemic myocardium, the change from the oxidative metabolism of free fatty acids to that of glucose is protective since it allows the ischemic myocardium to more efficiently utilize oxygen to generate the ATP necessary to preserve cellular transport systems and prevent cell death. However, the diabetic myocardium, because of impaired glucose transport into the myocardium, cannot metabolize this energy source and therefore there is decreased energy production and increased serum glucose levels [18]. 

Hyperglycemia leads to the formation of advanced glycation end products (AGE) and its cell surface receptor (RAGE) [19]. RAGE increases the inflammatory response by activating three key proinflammatory transcription factors which are normally suppressed by insulin: NFKB (nuclear factor), AP-1 (activated protein), and EGR-1 (early growth response) [20, 21]. Hyperglycemia also directly affects pathways responsible for changes in endothelial function, inflammation, and oxidative stress by altering the polyol pathway and increasing the synthesis of diacylglycerol which activates protein kinase-C [22, 23]. These changes in endothelial function contribute to decreased nitric oxide activity and increase production of superoxide radicals in diabetic internal mammary artery and saphenous vein grafts [24, 25]. Ultimately, this increased inflammatory response results in oxidative vascular stress which contributes to vascular thrombosis, plaque rupture, and impaired platelet function [26, 27]. These all contribute to reduced graft patency, recurrent ischemic events, and increased need for revascularization procedures in CABG patients with diabetes mellitus.

## 3. Beneficial Effects of Insulin in Ischemic Myocardium

Insulin reverses the harmful effects of hyperglycemia on vascular oxidative stress by increasing myocardial glucose uptake, diminishing the inflammatory response, and decreasing apoptosis. Insulin enhances myocardial glucose metabolism by facilitating glucose transport into the myocyte, inhibiting the release of free fatty acids, and augmenting aerobic metabolism by stimulating pyruvate dehydrogenase [28]. It acts as an anti-inflammatory agent by suppressing the proinflammatory transcription factors and NFKB, EGR-1, and AP-1 and reduces inflammatory mediators such as IL-6, TNF-alpha, ICAM-1, and E-selectin [21, 29, 30]. Insulin up, regulates the L-arginine nitric oxide pathway, thus promoting vasodilatation and enhancing endothelial function, improving platelet function by decreasing PA-1, and increasing prostacyclin release, and reducing apoptosis by increasing nitric oxide levels by a P-13 kinase dependent pathway [31, 32]. In clinical studies, insulin has been shown to decrease levels of free fatty acids following CABG, improves aerobic metabolism when added to cardioplegic solutions, and decreases levels of reactive oxygen species, adhesion molecules, and C-reactive protein [33–35]. 

## 4. Hyperglycemia Is Associated with Poor Outcomes in Patients with Acute Coronary Syndromes and CABG Surgery

Numerous studies have now shown that patients with acute coronary syndromes (ACS) who present with hyperglycemia are at a higher risk for death and in-hospital complications [36–43]. Capes and coworkers in a meta-analysis of 15 studies showed that the risk of in-hospital death in nondiabetic patients with ACS and an admission glucose >110 mg/dL was 3.9 times greater than in patients who were normoglycemic [36]. In diabetic ACS patients, a glucose ≥180 mg/dL had a 70% increased risk of in-hospital mortality compared to diabetic patients with normal admission glucose values. In 2,127 patients with ACS, Foo and coworkers showed a strong relationship between higher glucose levels and increased rates of both left ventricular failure and cardiac death [37]. Meier and coworkers demonstrated larger infarct size and increased long-term mortality in all ACS patients (both diabetic and non-diabetic) admitted with hyperglycemia [38]. It appears that the increased risk of mortality in ACS patients presenting with hyperglycemia is not restricted to patients with diabetes mellitus and may be higher in non-diabetics [39, 40]. In the Cooperative Cardiovascular Project which examined 141,680 elderly ACS patients, 30 day and one-year mortality was directly related to the level of hyperglycemia and was not limited to patients known to have preexisting diabetes mellitus [41]. In the Clinical Trial of Reviparin and Metabolic Modulation in Acute Myocardial Infarction Treatment and Evaluation-Estudios Clinicos Latino America (CREATE-ECLA) involving patients admitted with ST segment elevated MIs, mortality increased from 6.6% in patients with the lowest admission glucose levels to 14% in those with the highest levels [42]. In the Hyperglycemia: Intensive Insulin Infusion in Infarction (HI-5) study, patients with ACS presenting with glucose levels >140 mg/dL had a significantly higher six-month mortality rate [43]. Hyperglycemia following hospital admission has been shown to be even more important in predicting adverse outcomes in ACS patients, both in diabetic and non-diabetic patients [44–46]. 

Several studies have demonstrated that hyperglycemia is also associated with increased morbidity and mortality in both diabetic and non-diabetic patients undergoing CABG surgery. Donst and coworkers reviewed the outcomes of 6,280 patients undergoing cardiac procedures and found that patients with higher peak glucose levels (>360 mg/dL) during CABG had a higher incidence of morbidity and mortality irrespective of whether they were known to have diabetes mellitus [47]. Fish and coworkers found that elevated postoperative serum glucose levels (>250 mg/dL) were associated with a ten-fold increase in complications [48]. Similar findings of increased postoperative morbidity of elevated serum glucose levels were noted by McAlister et al., Gandhi, Székely et al., Imran et al., and Duncan et al. [49–52]. Henderson and coworkers found that CABG patients with impaired fasting glucose levels had doubled the one-year mortality rate [53]. These studies strongly suggest that patients with and without diabetes mellitus with elevated glucose values during ACS and in the perioperative period following CABG and cardiac surgery have increased morbidity and mortality.

## 5. Role of Glucose-Insulin-Potassium Solutions in the Treatment of the Ischemic Myocardium

One of the earliest concepts of insulin to treat coronary artery disease was as a component of glucose-insulin-potassium (GIK) solutions. In 1965, Sodi-Pallares and colleagues used GIK in patients experiencing acute myocardial infarctions and found that it limited electrocardiographic changes [54]. However, other trials failed to show a survival benefit in these patients [55]. Lazar and coworkers found that in an experimental porcine model which simulated surgical revascularization of acutely ischemic myocardium, hearts treated with GIK had less myocardial tissue acidosis, better preservation of regional wall motion, and the least tissue necrosis [56]. In a subsequent clinical trial in non-diabetic patients undergoing CABG surgery, Lazar and coworkers demonstrated that patients receiving GIK in the early postoperative period had higher cardiac indices, required less inotropic support, gained less weight, spent less time on the ventilator, had a lower incidence of atrial fibrillation, shorter ICU, and hospital length of stays [57]. These beneficial effects were realized despite the fact that GIK patients had significantly higher mean postoperative glucose levels (240 mg/dL versus 145 mg/dL; *P* < 0.02). No attempt was made to control serum glucose in this cohort of non-diabetic patients. Quinn and coworkers conducted a similar trial in CABG patients in which supplemental insulin was administered to keep serum glucose <270 mg/dL [58]. Similar to the results seen by Lazar and coworkers, GIK patients had significantly higher cardiac indices, a lower incidence of low cardiac output syndrome, and less biochemical and electrocardiographic evidence of myocardial injury. 

## 6. Modification of GIK Solutions in Diabetic CABG Patients

Patients known to have diabetes mellitus were excluded from GIK trials. Subsequently, studies were undertaken to modify the glucose concentration in GIK such that it could be used in diabetic patients. The Diabetes Mellitus, Insulin Glucose in Acute Myocardial Infarction (DIGAMI) trial involved 620 patients with an acute MI [59]. These patients were randomized to receive an i.v. GIK infusion in which the concentration of glucose was significantly reduced. Patients treated with this modified GIK solution had a 30% reduction in mortality over one year which persisted for a mean of 3.5 years [60]. This study prompted Lazar and coworkers to determine whether a similar modified GIK solution using more insulin and more glucose (500 mL/D5W + 80 units regular insulin + 40 mEq Kcl) designed to keep serum glucose <180 mg/dL would limit ischemic damage in diabetic CABG patients [61]. In this trial, 141 diabetic patients were randomized to receive either the modified GIK solution or sliding scale insulin coverage designed to maintain serum glucose <250 mg/dL. GIK patients achieved better glycemic control in the operating room in the initial 12 hours following surgery. They had significantly lower lactate and serum free fatty acid levels. This contributed to higher cardiac indices and less need for inotropic support in GIK-treated patients. Although there were no mortalities in either group, GIK patients had less morbidity. They had a significantly lower incidence of wound infections and atrial fibrillation, and spent significantly less time on the ventilator. This contributed to a shorter hospital length of stay (6.5 versus 9.2 days; *P* = 0.0003). Furthermore, following five years, GIK patients had a significantly lower incidence of recurrent ischemia, a lower angina class, and a significantly increased survival. This study highlighted the importance of continuous insulin infusions as opposed to intermittent subcutaneous insulin to achieve glycemic control in diabetic CABG patients. Furthermore, it showed that tight glycemic control could not only improve perioperative outcomes but also increase long-term survival and reduce recurrent ischemic events.

Other clinical trials demonstrated that insulin infusions alone, without glucose substrate, could improve perioperative outcomes in CABG patients with and without diabetes mellitus in the presence of hyperglycemia. One of the earliest studies showing the benefits of tight glycemic control during cardiac surgery was reported by Furnary and coworkers [62]. In a study cohort involving 3,554 patients undergoing CABG surgery from 1987 to 2001; continuous insulin infusions using the Portland Protocol to keep serum glucose within 100–150 mg/dL resulted in significantly lower mean glucose levels that could not be obtained with intermittent subcutaneous insulin therapy. This resulted in a 50% reduction in operative mortality in diabetic CABG patients. From 200 to 2005, an additional 1,980 patients were managed using the Portland Protocol [63]. Furnary and coworkers assessed glycemic control in these patients using a formula entitled “3-BG.” This consisted of the average of all glucose values obtained on the day of surgery and the first and second postoperative days. An increase in 3-BG was found to be an independent predictor of perioperative mortality, deep sternal wound infections, and hospital length of stay. An increased 3-BG was also associated with a significant increase in blood transfusions, new onset atrial fibrillation, and low cardiac output syndrome. 

Sternal wound infection remains a significant source of morbidity and mortality in the diabetic CABG patient and is more likely to occur when serum glucose exceeds 200 mg/dL in the postoperative period [64]. Kerr and coworkers found in 1,585 diabetic CABG patients that the incidence of sternal wound infections rose from 1.3% to 6.7% when glucose values exceeded 250 mg/dL [65]. Maintaining patients on a continuous insulin infusion with mean glucose values of 100–150 mg/dL significantly decreased the incidence of sternal infection. Hurska and coworkers demonstrated that using continuous insulin infusions to maintain glucose levels between 120 to 160 mg/dL significantly decreased the incidence of wound infections in diabetic CABG patients [66]. Further insight into the mechanism for the favorable effect of insulin infusions on wound infections was provided by Rassias and coworkers in a prospective randomized study in diabetic cardiac surgical patients [67]. Neutrophil phagocytic activity was better preserved in patients on a continuous insulin drip than those who received only an intermittent insulin bolus to treat perioperative hyperglycemia. Improved phagocytic function in the neutrophils of diabetic cardiac surgical patients may be the mechanism responsible for the reduced incidence of wound infections seen in these patients.

The importance of tight glycemic control in CABG patients was also noted by Van den Berghe and coworkers involving 1,048 ventilated patients admitted to a surgical ICU [68]. Patients were randomized to either a conventional therapeutic group in which insulin was administered only if serum glucose exceeded 250 mg/dL and an intensive group in which a continuous insulin infusion was used to maintain glucose levels between 80 to 110 mg/dL. Intensive insulin therapy resulted in a significant reduction of mortality (10% versus 20%; *P* = 0.005), exclusively in those patients requiring ≥5 days of ICU care with multiorgan failure and sepsis. Cardiac surgical mortality was reduced in those patients requiring ≥3 days of ICU care. Intensive glycemic control had no effect on morbidity and mortality in those patients spending ≤3 days in the ICU. D'Alessandro and coworkers sought to correlate tight glycemic control with expected EuroScore outcomes in diabetic CABG patients in an attempt to identify those patients who might benefit most from tight glycemic control [69]. In patients achieving tight glycemic control and continuous insulin infusions, observed mortality was significantly lower than expected (1.3% versus 4.3%, *P* = 0.01). In contrast, there was no difference between observed and expected mortality in the group without tight glycemic control. The benefit of tight glycemic control was the greatest in higher risk patients; those with a EuroScore >4 (2.5% observed versus 8.0% expected; *P* = 0.03). The authors concluded that diabetic patients with the highest risk tend to benefit most from tight glycemic control. 

## 7. The Role of Tight Glycemic Control in Nondiabetic CABG Patients

Will tight glycemic control benefit nondiabetic patients undergoing CABG surgery? Butterworth and coworkers studied the effect of tight glycemic control on 381 nondiabetic patients undergoing isolated CABG surgery [70]. In this prospective, randomized trial, one group received a continuous insulin infusion to maintain intraoperative glucose levels <100 mg/dL. The other group received no insulin coverage. Although intraoperative glucose levels were significantly lower in the patients receiving an insulin infusion, there was no difference in mortality or morbidity between the two groups. Hence, in this study of nondiabetic CABG patients, tight glycemic control failed to improve clinical outcomes. Ghandi and coworkers prospectively randomized 400 elective CABG patients to a continuous insulin group to maintain serum glucose between 80 to 100 mg/dL or a conventional group which used intermittent boluses of i.v. insulin to keep serum glucose <200 mg/dL [71]. The incidence of diabetes mellitus was 20% in both groups. There was no significant difference in morbidity, mortality, or length of hospital stay between the two groups, although there was a tendency for more deaths and strokes in the intensive insulin group. There were, however, several limitations in the study. Both diabetic and nondiabetic patients were included in the study and both groups received intensive insulin therapy in the immediate postoperative period so that a clear distinction between tight glycemic control versus intermittent insulin therapy could not be made. Furthermore, both groups averaged serum glucose <180 mg/dL in the postoperative period so that the patients without a continuous insulin infusion achieved the same degree of glycemic control as those with only intermittent insulin coverage. 

The benefits of tight glycemic control in nondiabetic patients are clouded by the fact that a growing number of patients undergoing CABG surgery have nondiagnosed diabetes mellitus, and abnormal glucose tolerance, with a metabolic syndrome. These patients may exhibit abnormal glucose levels only in the perioperative period. Previous studies have shown that nondiabetic patients with glucose levels >250 mg/dL who did not receive insulin therapy have increased hospital mortality [50]. Hence, as is discussed below, it is important that all cardiac surgical patients with elevated perioperative glucose values be treated with continuos insulin infusions, irregardless of whether a preoperative diagnosis of diabetes has or has not been established. 

## 8. Management of Hyperglycemia in the Perioperative Period

Achieving glycemic control in the perioperative period requires a multidisciplinary approach which includes representation from nursing, anesthesiology, pharmacy, surgery, and endocrinology [72]. At our own institution, we formed a Perioperative Glycemic Control Committee which has resulted in serum glucose levels <180 mg/dL in the first 48 hours in 94% of all cardiac surgery patients [73]. 

Glycemic control in the cardiac surgical patient is best achieved with strategies that are instituted in the preoperative period. All patients should have a hemoglobin A1c (HbA1c) drawn prior to surgery. The HbA1c is an indication of glycemic control in the 6–8 weeks prior to surgery. Adequate glycemic control is associated with an HbA1c < 7% [74]. Obtaining an HbA1c prior to surgery in diabetic patients and those patients at risk for postoperative hyperglycemia helps to optimize glycemic control in those patients with elevated HbA1c levels. In general, oral hyperglycemic medication should not be taken in the 12 hours prior to surgery. Patients who are taking insulin and who are admitted on the day of surgery should continue their basal insulin dose and hold their nutritional insulin. NPH insulin should be reduced by one-half or one-third prior to surgery to avoid hypoglycemia. Intravenous insulin is the preferred method of insulin delivery to achieve rapid and effective glycemic control in hospitalized patients who are hyperglycemic prior to surgery [75]. It is important to identify all patients with abnormal renal function since the risk for hypoglycemia is increased in all these patients [76–78]. 

During surgery, it is important to realize that insulin resistance increases but then rapidly decreases in the postoperative period. This results in an intraoperative rise in insulin requirements followed by a rapid fall in the immediate postoperative period. This is due to hypothermia, the increased glucose load associated with cardioplegia delivery, the glucose used to prime the cardiopulmonary bypass circuit, and the need for inotropic support [79]. Following discontinuation of cardiopulmonary bypass, when these factors are no longer present, insulin requirements decrease rapidly and if unrecognized, severe hypoglycemia can result [80]. Therefore, it is necessary to check glucose levels prior to leaving the operating room and make the appropriate reduction in insulin delivery. Glucose levels should be monitored every 30–60 minutes in the operating room, and as often as every 15 minutes during periods of rapid fluctuation, such as during cardioplegic infusions and systemic cooling and rewarming. It is our policy to obtain an endocrinology consult in all patients who require intraoperative insulin infusions for hyperglycemia since a significant percentage of these patients are ultimately found to have diabetes mellitus. 

In the ICU, all patients should have serum glucose values ≤180 mg/dL as recommended by the STS guidelines [81]. Patients who require ≥3 days in the ICU because of ventilatory dependency, the need for inotropes, intraaortic balloon pump or left ventricular assist device support, anti-arrhythmics, dialysis, or continuous venovenous hemofiltration should receive continuous insulin infusions to keep blood glucose <150 mg/dL regardless of their diabetic status.

Multiple protocols for ICU continuous insulin infusions have been established [82–84]. Recently, computer-based algorithms have become commercially available to assist the nursing staff in adjusting insulin infusion rates [85, 86]. Although studies have shown that computer-based algorithms have been associated with tighter glucose control, there have been no reported differences in the frequency of hypoglycemic events, length of ICU and hospital stay, or mortality with these algorithms; their use depends on physicians' preferences and cost considerations [87–89]. 

The following are the current recommendations of the Society of Thoracic Surgery regarding blood glucose management during adult cardiac surgery [81].

- All patients with diabetes undergoing cardiac surgical procedures should receive an insulin infusion in the operating room and for at least 24 hours postoperatively to maintain serum glucose levels <180 mg/dL (Class I; Level of Evidence B).
- An HbA1c level should be obtained prior to surgery in patients with diabetes, and those patients at risk for postoperative hyperglycemia to characterize the level of postoperative glycemic control (Class I; Level of Evidence C).
- Glucose levels >180 mg/dL that occur in patients without diabetes only during cardiopulmonary bypass may be treated initially with a single intermittent dose of i.v. insulin as long as the levels remain <180 mg/dL. However, in those patients with persistently elevated glucose (>180 mg/dL) after cardiopulmonary bypass, a continuous insulin drip should be instituted (Class I; Level of Evidence B).
- Patients with and without diabetes with persistently elevated serum glucose (>180 mg/dL) should receive i.v. insulin infusions to maintain serum glucose <180 mg/dL for the duration of their ICU care (Class I; Level of Evidence A).
- All patients who require ≥3 days in the ICU because of ventilatory dependency requiring the need for inotropes, intraaortic balloon pump or left ventricular assist support, antiarrhythmics, dialysis, or continuous venovenous hemofiltration should have a continuous insulin infusion to keep blood glucose ≤150 mg/dL, irregardless of their diabetic status (Class I; Level of Evidence B).

### 8.1. Glucose Monitoring in the ICU

In order to avoid wide fluctuations in glucose levels, it is imperative that they be frequently monitored in the ICU. Our CII follows the common practice of obtaining hourly glucose values until stable targeted blood glucose levels have been achieved [90, 91]. Most patients have either an arterial or central venous monitoring line that allows for painless blood sampling. When there is anticipation of an inotrope or a dextrose solution causing rapid hyperglycemia, glucose values may be obtained every 30 minutes so that the target glucose level can be maintained. 

**Figure 1:**
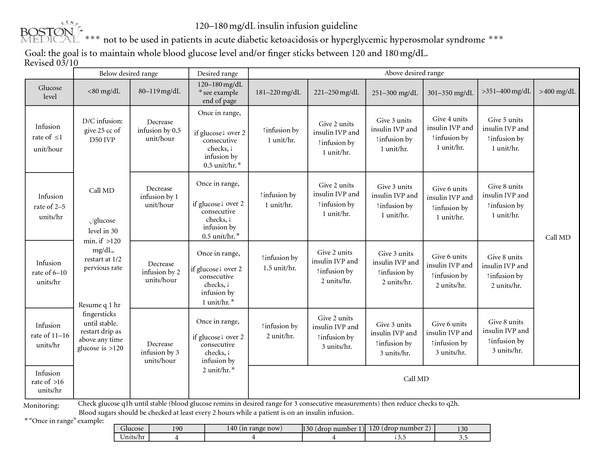
Continuous insulin infusion protocol.

Unfortunately, the accuracy of most hand-held glucose meters is far from optimal [92]. There is an accepted variance between meter readings and central laboratory results (allowed to be up to 20% by FDA regulations), which can potentially lead to inappropriate therapy [93, 94]. Many patient factors are known to affect the accuracy of the POC testing including pH changes, oxygenation status, and low hematocrit [92, 95]. Given these factors, all patients in the ICU have blood glucose levels determined by the central laboratory every 2 to 4 hours in the early postoperative period, and twice daily for up to 2 days. All glucose levels <70 mg/dL or >300 mg/dL are verified with blood samples sent to the central laboratory (Figure 1). 

### 8.2. Transition to SC Insulin Therapy

Transitioning the patient to SC insulin therapy is the most difficult of all the perioperative stages in terms of reliably maintaining adequate glycemic control. While many institutions have adopted a prolonged course of CII for up to three days postoperatively, hence ensuring glycemic control at least in the fasting state, this is not possible in all hospitals and depends largely on nursing and hospital support. In our hospital, CII therapy in the step down unit is used rarely and reserved for patients who continue to require over 3 units per hour of insulin in the fasting state or 6 units per hour while receiving nutrition despite being otherwise stable for step down care. In all other patients who do not require continued ICU care, it is usually possible to maintain and achieve excellent glycemic control off CII by a combination of long and rapid acting SC insulin dosed according to the insulin infusion requirements. We and others have found that using a consistent mathematical formula allows for a safe and effective transition from CII to a SC insulin regimen [96, 97]. 

The patient is ready to be transitioned to a scheduled basal insulin regimen when they meet the following criteria.

1. A stable intravenous insulin infusion rate is maintained for at least 4 hours in the fasting state.
2. The patient is extubated and is off pressor agents.
3. The patient is ready to receive oral, enteral, or parenteral nutrition.

These guidelines are followed and directly implemented by the Inpatient Diabetes consultative service, which serves to centralize direction of the insulin therapy during the postoperative period. While many patients with normal preoperative HbA1C levels may require CII for less than 24 hours, we find that most patients requiring at least 1 U/hour require a calculated transition to SC insulin on POD1 or POD2. A daily basal insulin requirement of 20 units (i.e., approximately 1 unit/hour) is significantly enough to justify a calculated insulin transition program.

The guideline steps are as follows.

**Figure 2:**
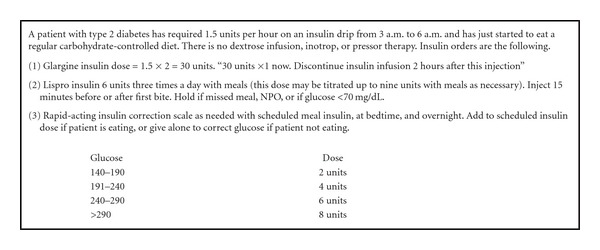
Example transition from continuous insulin infusion to subcutaneous insulin therapy.

(1) If the insulin rate exceeds 3 units per hour in the fasting state or 6 units per hour while on continuous nutrition, delay transition from CII for 8–12 hours. High insulin requirements may be indicative of the patient's preoperative requirements; however, it could be an early sign of infection or low cardiac output state. 

(2) If renal function becomes unstable during the first 24–48 hours postoperatively, as indicated either by reduced urine output or a rise in serum creatinine, continue CII until renal function stabilizes, as insulin requirements can change dramatically in the presence of acute renal dysfunction.

(3) When transitioning patients off CII, disregard outpatient regimens in the first 48 hours following surgery and do not start any oral or noninsulin injectable agents. Use the overnight infusion rate (fasting rate) for the transition to the basal insulin dose and extrapolate to 24 hours. Eighty percent of this extrapolated dose is given as basal insulin (e.g., glargine × 1 dose) and the insulin infusion is discontinued two hours later. Stated in another way, take the most stable overnight, fasting, insulin rate (e.g., 2 units/hour between 4 and 6 AM), and multiply by 20 (2 × 20 = 40). Administer this dose (e.g., order “40 units × 1 now”) and discontinue the insulin infusion 2 hours later.

(4) Schedule nutritional insulin for all patients as long as they are taking in nutrition, which is the case for >90% of patients in our experience. For patients ordered to have three carbohydrate-consistent meals each day, a rapid-acting insulin (Table 2) is administered within 15 minutes of the first bite of food. The total daily premeal dose is estimated based on the basal dose and split into three equivalent doses (e.g., per example above, 20/3 = approximately 6 units per meal). For patients eating poorly, reduce the meal insulin dose empirically by 20–30%. For patients receiving continuous enteral nutrition, rapid analog insulin is dosed every 4 hours, or regular insulin is dosed every 6 hours, to accommodate the food. Those patients on parenteral nutrition require insulin inside the bag and these dosing strategies have been previously reviewed [98, 99]. 

(5) Schedule fingersticks for premeal, bedtime, and at 2 a.m. for the first 2 days postoperatively, and use these values to modify the insulin dose.

(6) Redose basal insulin dose in 24 hours and daily. Most patients require a 20–30% reduction of the total insulin daily dose on the day following the transition, and sometimes each day thereafter, as insulin sensitivity improves. This downtitration depends largely on the patient's diabetes status and outpatient diabetes regimen. Many patients without diabetes or with well-controlled type 2 diabetes do not require basal insulin by postoperative day 3. While patients with type 1 diabetes will require basal insulin daily, the dose requirement is also expected to fluctuate during recovery (Figure 2). 

### 8.3. Glycemic Control Following-ICU

Our goal during the non-ICU phase of the patient's hospital stay is to coordinate optimal diabetes care while adhering to SCIP, STS, and the American Association of Clinical Endocrinology guidelines.

1. A target blood glucose level <180 mg/dL should be achieved in the postprandial state.
2. A target blood glucose level between 100 and 140 mg/dL should be achieved in the fasting and premeal states after transfer to the floor.

The best method to achieve consistent glycemic control in clinically stable patients with diabetes is with scheduled basal/bolus insulin therapy. This is done best with SC insulin that combines long or intermediate-acting insulin with rapid-acting insulin dosed simultaneously with nutritional intake. The dosing must take into account the patient's food intake and glucose levels. This must be adjusted in cooperation with nursing since many patients will have no experience with insulin therapy. Orders for rapid-acting prandial insulin must include instructions to withhold the insulin if the patient is not able to eat. Orders for long-acting basal insulin should include instructions not to withhold a dose if the patient has a normal glucose or is not eating, since this insulin should be dosed to support insulin needs in the fasting state.

### 8.4. Resuming Oral and Noninsulin Agents

Although noninsulin agents have not been studied in the hospital setting, we have found that some patients with type 2 diabetes who use oral medications can start these agents on POD3 in the hospital if they have resumed a normal diet and their glucose is within the target range. Insulin doses often have to be reduced or eliminated in some cases, once these oral agents are initiated. Often the main goal of restarting home oral agents is to ensure tolerability and safety in a patient who has achieved good control in the hospital postoperatively, is medically stable, and is expected to require at least another day in hospital. Sulfonylureas (glipizide, glyburide, and glimepiride) and short-acting insulin secretagogues (repaglinide, nateglinide) should be started slowly and based on the patient's appetite. Metformin should not be restarted until the patient is documented to have normal renal function. Thiazolidinediones (pioglitazone, rosiglitazone) should be avoided in all patients with congestive heart failure. Incretin mimetics or potentiators (oral sitagliptin and SC exenatide) can be resumed in most patients by POD3.

### 8.5. Preparation for Hospital Discharge

Any patient, with or without diabetes, who requires insulin therapy in the perioperative period is evaluated by the Inpatient Diabetes Service to determine the best plan for glucose management at home. The preoperative HbA1c level will help to determine and guide recommendations for therapy upon discharge [100, 101] (Table 1). We require all patients undergoing CT surgery to have an HbA1c included in the preoperative lab assessment in order to avoid having it checked postoperatively, when the test may be inaccurate. The patient's postoperative course will determine the resumption of outpatient medications. For example, patients receiving metformin or sulfonylurea may require discontinuation if they develop renal insufficiency. Similarly, patients will have to discontinue thiazolidinediones if they develop congestive heart failure, fluid overload, or suffer a decrease in ejection fraction. It is important to communicate these changes to the patient's local endocrinologist or primary physician. Patients and their physicians must be made aware of any changes that are instituted in their medications and insulin dosages postoperatively to avoid any adverse events, especially severe hypoglycemia.

It is not uncommon for a patient who has not been diagnosed with diabetes mellitus to become hyperglycemic and require insulin therapy in the perioperative period. This can represent either transient “stress hyperglycemia” due to the metabolic syndrome or previously undiagnosed diabetes mellitus. Although stress hyperglycemia resolves as the acute illness or surgical stress abates, it is important to identify and track patients as 60% of patients admitted with new hyperglycemia had confirmed diabetes at 1 year [102]. For those patients with a preoperative HbA1c < 6%, lifestyle counseling which includes diet and exercise alone may be sufficient. They should receive followup HbA1c and glucose levels by their local physician. For those patients with an HbA1c between 6 and 6.5%, follow-up testing for diabetes as well as institution of lifestyle changes is necessary. Patients discharged with a new diagnosis of diabetes must have followup with a physician to address the medication and lifestyle changes instituted in the postoperative period. Discharge planning regimens are summarized in Table 1. Completing the discharge treatment plan with the patient and achieving their compliance and cooperation is key to maintaining glycemic control following discharge.

## 9. Optimal Glucose Levels for CABG Surgery: Aggressive versus Moderate Control

Based on the data presented, it is now accepted that glycemic control improves short- and long-term outcomes in CABG patients with diabetes mellitus and those nondiabetics who exhibit perioperative hyperglycemia. However, the optimal target for serum glucose levels in the perioperative period is unknown. All studies have shown that maintaining serum glucose levels <180 mg/dL reduces morbidity and mortality, the effects of more aggressive control on clinical outcomes are less clearly defined. Recent trials in both ICU and non-ICU patients have shown that more aggressive glycemic control may actually increase mortality from cardiovascular disease and increase episodes of hypoglycemia [103–111]. In a study by Van den Berghe and coworkers, in patients who received aggressive insulin therapy to maintain serum glucose ≤110 mg/dL, cardiac surgical mortality was only reduced in those patients receiving more than 3 days of ICU care and in those with multiorgan failure and sepsis [68]. In a subsequent study of aggressive insulin therapy in critically ill, nonsurgical ICU patients by the same authors, there was no difference in hospital mortality between aggressive and moderate control [112]. Two additional medical ICU trials failed to show any improvement with aggressive glucose management and had to be discontinued because of increased episodes of hypoglycemia [105, 113]. In the Normal Glycemia in Intensive Care Evaluation Survival Using Glucose Algorithm Regulation trial, patients randomized to aggressive glycemic control (81–108 mg/dL) had a significantly higher mortality and increased episodes of hypoglycemia [103]. The excess deaths were predominantly from cardiovascular causes. Two large medical trials also failed to support aggressive glycemic control to improve mortality from cardiovascular disease [107, 109]. In order to determine the effects of more aggressive glycemic control in diabetic patients during CABG surgery, Lazar and coworkers prospectively randomized patients to either an aggressive (90–120 mg/dL) or moderate (120–180 mg/dL) protocol [114]. There was no difference in the incidence of a 30-day mortality, myocardial infarction, neurological events, deep sternal infections, or atrial fibrillation between the groups. Patients with aggressive control had a higher incidence of hypoglycemic events but this did not result in any clinical sequelae. Aggressive glycemic control did not result in any further improvement in clinical outcomes that could not be achieved with more moderate control. These results are consistent with those of Bhamidipati and coworkers who showed that moderate glycemic control (120–179 mg/dL) in diabetic CABG patients were associated with the least amount of morbidity and mortality [115]. The American College of Physicians now recommends achieving a more moderate glucose level of 140–200 mg/dL in surgical and medical intensive care unit patients [116]. 

## 10. Conclusions

Hyperglycemia which occurs during CABG and cardiac surgery increases perioperative morbidity and mortality and results in decreased long-term survival and recurrent ischemic events. Maintaining serum glucose ≤180 mg/dL with continuous insulin infusions in patients with and without diabetes mellitus reduces morbidity and mortality, lowers the incidence of sternal wound infections, reduces hospital length of stay, and enhances long-term survival. Patients who require >3 days of ventilatory support or develop sepsis or multiorgan failure should have serum glucose levels <150 mg/dL. More aggressive glycemic control (80–120 mg/dL) in the absence of these complications appears to offer no benefits and does not improve clinical outcomes.

### 10.1. Future Areas of Study

Important issues in the management of hyperglycemia during cardiac surgery remain to be elucidated. Studies will be needed to answer ongoing issues regarding perioperative glycemic control.

(1) What is the optimal level of glycemic control and which, if any, specific time period is most crucial for maintaining glycemic control? Is glucose management in the operating room more important than in the ICU? Our own experience and those of others seem to suggest that the ICU period is the most important period which determines the effectiveness of glycemic control on clinical outcomes [61, 68]. However, prospective, randomized trials will be necessary to answer this question.

(2) Is the level of glucose achieved as important as the amount of insulin delivered? Our studies would suggest that the level of glucose that is achieved is more important than the total amount of insulin delivered [114]. In CABG patients receiving larger amounts of insulin, as long as serum glucose was <180 mg/dL, there was no additional improvement in clinical outcomes.

 (3) What is the importance of preoperative HbA1c levels? Should patients with elevated HbA1c levels have their surgery delayed to minimize perioperative complications? Halkos and coworkers have shown that preoperative elevation of HbA1c levels are associated with both increased short- and long-term mortality following CABG surgery [117, 118]. However, in this study, glycemic control designed to keep serum glucose <180 mg/dL was not routinely practiced. In a prospective, randomized trial, Lazar and coworkers sought to determine whether preoperative HbA1c can predict postoperative complications in diabetic patients following CABG surgery when perioperative glycemic control (glucose < 180 mg/dL) was achieved [119]. In this study involving 167 CABG patients, the level of preoperative HbA1c was not predictive of a 30-day mortality, morbidity, or length of stay when glycemic control was achieved. This suggests that strategies which optimize glycemic control during CABG surgery may negate the effects of poor glycemic control prior to surgery. A larger study cohort will be necessary to determine whether these observations will continue to be true and whether surgery should be delayed in patients with higher HbA1c values.

(4) What is the optimal method to measure glucose values in the perioperative period? As noted previously, the accuracy of hand-held glucometers is not optimal and there is up to 20% variation between readings of glucometers and the central laboratory [92–94]. Another option is continuous glucose monitoring devices which provide a continuous glucose value. However, it is not known how accurate these devices will be during cardiac surgical procedures during periods of hypothermia, inotropic support, vasoconstriction, and vasodilation. Instantaneous readings may not result in improved glycemic control and may accentuate the frequency of hypoglycemia because of the delay in the insulin response. Larger, prospective studies will be needed to define the role of these devices in achieving glycemic control in cardiac surgical patients.

## Figures and Tables

**Table 1:** Suggested use of the HA1c during hospitalization for discharge planning for hyperglycemic patients.

	Unknown diabetes	Known diabetes	Followup
HA1c < 6.5%*	Assess diabetes risk factors. Counseling and outpatient screening within 3 months		
HA1c 6.5–7%* and insulin requirement < 0.4 units/kg/day	Counseling and outpatient screening within 3 months ± pharmacologic prevention**	Assess for hypoglycemia risk. Continue prehospital regime unless new safety concerns.	Communicate recommendation to outpatient providers. Address need for referral to multidisciplinary care for diabetes treatment or prevention
HA1c 6.5–7%* and insulin requirement ≥ 0.4 units/kg/day	Counseling and *initiation* of appropriate diabetes treatment plan		
HA1c > 7%*	Counseling and initiation of appropriate diabetes treatment plan	Consider transient effect of subacute illness (e.g., angina) prior to hospitalization on HA1c. Consider advising augmentation of outpatient regimen to target <7%	

Adapted from Supplement to ACP Hospitalist. December 15, 2009. *Note, the A1c is inaccurate after blood transfusion and in severe anemia, or in high or low red blood cell turnover states. **Metformin or acarbose.

**Table 2:** Pharmacokinetics of insulin preparations.

Type of insulin	Onset	Peak (hours)	Duration (hours)
Rapid analogs (lispro, aspart, glulisine)	5–15 minutes	1-2	4–6
Short (regular)	30–60 minutes	2-3	6–10
Intermediate (NPH*)	2–4 hours	4–10	12–18
Long (glargine)	2–4 hours	Flat	20–24
Long (detemir)	2 hours	Flat	6–24

*NPH: Neutral Protamine Hagedorn.
